# Evaluation of COVID-19 Vaccine Attitudes among Arab American Healthcare Professionals Living in the United States

**DOI:** 10.3390/vaccines9090942

**Published:** 2021-08-24

**Authors:** Anita Shallal, Evi Abada, Rami Musallam, Omar Fehmi, Linda Kaljee, Ziad Fehmi, Suma Alzouhayli, Deema Ujayli, Doreen Dankerlui, Seongho Kim, Michele L. Cote, Vijaya Arun Kumar, Marcus Zervos, Rouba Ali-Fehmi

**Affiliations:** 1 Division of Infectious Disease, Henry Ford Hospital, 2799 W. Grand Blvd, CFP 303, Detroit, MI 48202, USA; ashalla2@hfhs.org (A.S.); mzervos1@hfhs.org (M.Z.); 2 Department of Pathology, Wayne State University School of Medicine/Detroit Medical Center, 3990 John R. Rd, Detroit, MI 48201, USA; gs5839@wayne.edu; 3 Wayne State University School of Medicine, 4201 St Antoine, Detroit, MI 48201, USA; rami.musallam@wayne.edu (R.M.); suma.alzouhayli95@gmail.com (S.A.); 4 The University of Michigan College of Literature, Science, and the Arts, 101 N Main St, Ann Arbor, MI 48104, USA; ofehmi@umich.edu (O.F.); zfehmi@umich.edu (Z.F.); 5 Global Health Initiative, Henry Ford Health System, One Ford Place, 1E, Detroit, MI 48202, USA; lkaljee1@hfhs.org (L.K.); ddanker1@hfhs.org (D.D.); 6 Michigan State University College of Human Medicine, 965 Fee Rd A110, East Lansing, MI 48824, USA; ujaylide@msu.edu; 7 Biostatistics Core, Karmanos Cancer Institute, Department of Oncology, Wayne State University School of Medicine, 4100 John R. St, Detroit, MI 48201, USA; kimse@karmanos.org; 8 Population Sciences and Disparities Research, Karmanos Cancer Institute, Department of Oncology, Wayne State University School of Medicine, 4100 John R. Rd, Detroit, MI 48201, USA; cotem@karmanos.org; 9 Department of Emergency Medicine, Wayne State University School of Medicine/Detroit Medical Center, 3990 John R. Rd, Detroit, MI 48201, USA; vkumar@med.wayne.edu

**Keywords:** COVID-19, vaccine hesitancy, vaccine confidence, vaccine attitudes, Arab Americans

## Abstract

Background: Vaccine hesitancy is the next great barrier for public health. Arab Americans are a rapidly growing demographic in the United States with limited information on the prevalence of vaccine hesitancy. We therefore sought to study the attitudes towards the coronavirus disease 2019 (COVID-19) vaccine amongst Arab American health professionals living in the United States. Methods: This was a cross sectional study utilizing an anonymous online survey. The survey was distributed via e-mail to National Arab American Medical Association members and Arab-American Center for Economic and Social Services healthcare employees. Respondents were considered vaccine hesitant if they selected responses other than a willingness to receive the COVID-19 vaccine. Results: A total of 4000 surveys were sent via e-mail from 28 December 2020 to 31 January 2021, and 513 responses were received. The highest group of respondents were between the ages of 18–29 years and physicians constituted 48% of the respondents. On multivariable analysis, we found that respondents who had declined an influenza vaccine in the preceding 5 years (*p* < 0.001) and allied health professionals (medical assistants, hospital administrators, case managers, researchers, scribes, pharmacists, dieticians and social workers) were more likely to be vaccine hesitant (*p* = 0.025). In addition, respondents earning over $150,000 US dollars annually were less likely to be vaccine hesitant and this finding was significant on multivariable analysis (*p* = 0.011). Conclusions: Vaccine hesitancy among health care providers could have substantial impact on vaccine attitudes of the general population, and such data may help inform vaccine advocacy efforts.

## 1. Introduction

Vaccine hesitancy is the next great barrier for public health officials and health care providers in the wake of the coronavirus 2019 (COVID-19) pandemic. Hesitant attitudes to vaccination are prevalent and may be increasing since the influenza pandemic of 2009 [1]. The term “vaccine hesitancy” is used to describe patients who contemplate delaying vaccines or are reluctant to receive vaccines, implying that the hesitant can be swayed toward or away from acceptance [2], this hesitancy can vary across vaccines. Public acceptance of a vaccine is required to maintain herd immunity and prevent outbreaks of vaccine-preventable illnesses [3]. The issue is of the utmost importance, to the extent that the World Health Organization named vaccine hesitancy one of the top 10 threats to global health in 2019 [4]. A significant barrier to vaccination is misinformation regarding the benefits, composition, and adverse effects of vaccination. Different populations experience vaccine hesitancy to a varied extent and this may influence hesitancy among healthcare workers from these populations. Identifying at risk populations can help guide public health campaigns that specifically targets these groups.

The factors that contribute to hesitancy can vary between communities including demographic, socioeconomic, and societal factors. There are racial differences in trust and confidence when an individual is in the vaccine decision-making process [5]. Arab Americans (AA) are a rapidly growing ethnic minority in the United States (US) with a growth rate of 72% from 2000–2010 [6]. Arab Americans are individuals with roots or origins from the 22 countries that constitute the “Arab world” who now live in the United States [7]. Attitudes to vaccines vary across geographical boundaries and little is known about the prevalence of vaccine hesitancy among AA living in the US. One recent analysis by Sallam reports that some of the lowest rates of vaccine acceptance are in the Middle East [8], and interestingly, vaccine acceptance is higher among Arabs living outside of their home country [9,10]. A recent study surveyed multiple populations from different Arab countries and noted that only 29.4% of respondents to a survey stated that they would be vaccinated [11], compared to approximately 68% of respondents in the US [12]. Another study showed that the rate of acceptance to the COVID-19 vaccine varies across Middle Eastern countries, from 35% in people from Iraq to 17% in individuals from Jordan [13]. While a number of societal factors may play a role in vaccine hesitancy in Arab counties, distrust for government and healthcare policies are commonly reported reasons [13,14].

Healthcare workers are a major source of information for COVID-19 vaccines. According to one survey, 36.4% of Arabs reported that their major source of information regarding the COVID-19 vaccines was obtained from medical doctors, scientists, and scientific journals [11]. Another study noted that 53.1% of respondents sourced information about the COVID-19 vaccines from medical websites, while 32.7% accessed information about the vaccine from social media [13]. Amongst healthcare providers from the Arab world, one survey noted the vaccine acceptance rate was as low as 26.7%, and acceptance varied between countries [10]. Recent literature also suggests that Arab-speaking physicians have higher receptivity of COVID-19 vaccines when compared to nursing and allied health professionals [15]. According to Arab-speaking healthcare providers, the most frequently chosen barrier to vaccine acceptance was fear of side effects [10]. As AA turn to their providers for recommendations, determining the extent of vaccine hesitancy among this subgroup of AA living in the US is crucial. This information could guide or influence public health outreach efforts to increase acceptance of the COVID-19 vaccine among AA as a whole.

Two distinctive organizations are recognized for specifically providing services for people of Arab descent living in the US. The first is the National Arab American Medical Association (NAAMA), an organization that acts as the voice for approximately 5000 AA healthcare professionals and promotes professional development and cultural identity through educational, philanthropic, and service activities [16]. NAAMA, which has 27 chapters across North America, also has a separate distinct mission of enhancing the careers of undergraduate and graduate students who are members of the association and aspiring to practice in the healthcare field [17]. These undergraduate and graduate students comprise NAAMA NextGen. The second is the Arab-American Center for Economic and Social Services (ACCESS), which is the largest and most comprehensive Arab community-based health and mental health center in North America. Established in 1971, ACCESS is considered a “one stop service center” for medical, public health, mental health, and environmental programs for this particular community [18]. ACCESS is located in southeast Michigan and caters to residents of Arab descent in the area. In addition, ACCESS employs hundreds of AAs, including healthcare providers. These organizations provide a unique opportunity to determine the prevalence and factors associated with vaccine hesitancy among AA healthcare professionals based in the United States. We therefore aimed to study AA healthcare workers’ attitudes surrounding the COVID-19 vaccine and to determine the factors associated with vaccine hesitancy and confidence.

## 2. Materials and Methods

### 2.1. Study Design

This was a cross sectional study utilizing an anonymous online survey. The survey platform utilized was SurveyMonkey (San Mateo, CA, USA). Adults aged 18 years or older and able to provide informed consent were recruited via e-mail to NAAMA members and ACCESS healthcare employees. A total of 4000 surveys were sent via e-mail from 28 December 2020, and the survey was closed on 31 January 2021.

### 2.2. Ethical Considerations

Informed consent from the participants was obtained and possible consequences of the study were fully explained. The study was determined to be non-human subjects research by the Institutional Review Boards of Henry Ford Health System and Wayne State University School of Medicine. The study also received a letter of support through the Research Review process at ACCESS. Emails for NAAMA and NAAMA NextGen members and ACCESS employees were obtained through approval by each independent Board of Directors. Participant privacy was achieved by use of anonymous surveys.

### 2.3. Survey Items

Survey items included demographic information such as age, gender, marital status, country of birth, time of immigration to the US, current regional location in the US, highest level of education, employment status, type of healthcare professional (categorized as physicians, nurses, allied healthcare professional, or student), and yearly gross household income. Allied health professionals included hospital administrators, case managers, medical assistants, researchers, scribes, pharmacists, dieticians, and social workers. To determine information about prior vaccine behaviors, influenza vaccination history and history of immunization deferral or rejection for participants’ children was assessed. Attitudes regarding COVID-19 and COVID-19 vaccines were then ascertained through items that included having personally known someone who contracted COVID-19, having personally known someone who died of COVID-19, plans to receive the COVID-19 vaccine, and mandating vaccination beliefs.

### 2.4. Statistical Methods

Categorical variables were summarized by count and frequency, and median and range were used to summarize continuous variables. Vaccine hesitancy was defined using the SAGE Working Group on Vaccine Hesitancy definition: “a delay in acceptance or refusal of vaccination despite availability of vaccination services” [19]. Hesitancy was defined as binary (Yes vs. No, with No used as reference) using the question “I plan to receive a COVID-19 vaccine”. We considered that a participant may hesitate to receive a COVID-19 vaccine if one answered, “will probably get the vaccine”, “will probably NOT get the vaccine”, “will definitely NOT get the vaccine”, and “don’t know/not sure”. Vaccine confidence was defined as those who answered, “will definitely get the vaccine” or “have already received the vaccine”. Univariable and multivariable logistic regression analyses were performed to see the associations between vaccine hesitancy (Yes vs. No, with No used as reference) and participant characteristics and attitudes toward the vaccine. We allowed the events per variable at least to be 5 [20] and further used Firth’s logistic regression to improve the accuracy of regression coefficients [21]. Initially, vaccine hesitancy was considered as an ordinal variable and univariable and multivariable ordinal logistic regression analyses were used. However, six covariates (age, marital status, country origin, education, health care professional, and knowing a COVID-19 person) violated the proportional odds assumption. To resolve this violation, we dichotomized vaccine hesitancy and used binary logistic regression analysis.

## 3. Results

### 3.1. Participant Characteristics

Of the 4000 surveys sent, we received 513 responses, corresponding to a participant response rate of 12.8%. The highest group of respondents were between the ages of 18 and 29 years (*n* = 216, 42%). Respondents over 70 years old constituted 7% (*n* = 36) of the total survey participants. Two hundred and seventy-one (53%) respondents were men and 238 (46%) were women. About half (261, 51%) of the respondents were married, while 230 (45%) were single. Nearly half of the respondents were born in the Middle East (*n* = 246, 48%), and *n* = 226 (44%) were born in North America. Of those who had immigrated to the US (*n* = 294), *n* = 218 (74%) had been in the US for 20 years or longer, and *n* = 76 (26%) had been in the US for less than 10 years.

More than half of the participants (*n* = 279; 54%) received a professional degree, and *n* = 169 (33%) received a bachelor’s degree or higher. Thirty-eight (7%) completed some college or university degree, and *n* = 26 (5%) received a high school diploma or less. The majority of participants (*n* = 317; 62%) were employed full or part time, *n* = 148 (29%) were students, *n* = 30 (6%) were retired and *n* = 17 (3%) were unemployed. Two hundred and forty-five (48%) respondents were physicians, *n* = 141 (27%) were students, *n* = 93 (18%) were allied health professionals, and *n* = 5 (1%) were nurses. Twenty-nine (6%) respondents declined to provide their employment information. About one half (51%) of the respondents had a yearly household income of over $150,000. A summary of these results is presented in Table 1.

### 3.2. Prior Vaccine Behaviors and COVID-19 Vaccine Attitudes

Table 2 describes attitudes and behaviors regarding other vaccinations as well as the COVID-19 vaccine. To ascertain prior vaccine behaviors, participants were asked questions about childhood immunizations and influenza vaccinations. The vast majority of participants (*n* = 443, 86%) received an influenza vaccine in the last 5 years. Among the *n* = 285 respondents with children, only *n* = 17 (5.9%) reported delaying prior childhood immunizations for reasons other than illnesses or allergies.

Specific to COVID-19, nearly all respondents (*n* = 486, 95%) reported that they personally knew someone who had contracted the infection and over half reported that they personally knew someone who had died of COVID-19 (*n* = 293, 57%). In our study population, *n* = 212 (41%) reported they already received the COVID-19 vaccine, while *n* = 176 (34%) reported they were definitely going to receive the vaccine. Sixty-five (13%) responded that they would “probably” receive the vaccine, *n* = 30 (6%) were “not sure” if they would receive the vaccine, while 6% (*n* = 30) would “probably NOT” or “definitely NOT” receive the vaccine. Respondents were divided on vaccine mandates, with 45% of respondents (*n* = 231) of the opinion that a COVID-19 vaccine should be mandated, while 162 (32%) did not think a COVID-19 vaccine should be mandated, and *n* = 118 (23%) respondents were unsure.

### 3.3. Univariable and Multivariable Analysis

Following our review, we found that respondents who identified as allied healthcare professionals were more likely to be vaccine hesitant on both univariate (odds ratio (OR): 7.975; 95% CI: 4.585–14.140; *p* < 0.001) and multivariable (OR: 2.434; 95% CI: 1.117–5.319; *p* = 0.025) analyses. Respondents who declined an influenza vaccine in the preceding 5 years were also more likely to be vaccine hesitant and this characteristic was significant on both univariate (OR: 12.967; 95% CI: 7.037–25.044; *p* < 0.001) and multivariable (OR: 8.896; 95% CI: 4.315–19.252; *p* < 0.001) analyses. However, respondents making over $150,000 annually were less likely to be vaccine hesitant compared with respondents who made less than $150,000 annually, and this finding was significant on both univariate (OR: 0.223; 95% CI: 0.139–0.349; *p* < 0.001) and multivariable (OR: 0.455; 95% CI: 0.243–0.838; *p* = 0.011) analyses.

When compared with women, we found that men were less likely to be vaccine hesitant on univariate analysis (OR: 0.461; 95% CI: 0.304–0.694; *p* < 0.001); however, this observation was not significant on multivariable analysis (OR: 0.963; 95% CI: 0.557–1.669; *p* = 0.893). In addition, married individuals were less likely to be vaccine hesitant compared with single individuals, and even though this observation was significant on univariate analysis (OR: 0.501; 95% CI: 0.327–0.764; *p* < 0.001), it did not hold true on multivariable analysis (OR: 1.939; 95% CI: 0.861–4.485; *p* = 0.111).

Respondents who were born in North America/Europe were also more likely to be vaccine hesitant compared with respondents born in North Africa/Middle East and this observation was significant on univariate analysis (OR: 1.876; 95% CI: 1.249–2.832; *p* = 0.002), but not on multivariable analysis (OR: 0.999; 95% CI: 0.267–3.390; *p* = 0.998). Respondents who personally did not know someone who had contracted COVID-19 were more likely to be vaccine hesitant compared to people who personally knew someone who had contracted the disease. However, while this observation was significant on univariate analysis (OR: 3.099; 95% CI: 1.419–6.730; *p* = 0.005), it did not hold true on multivariable analysis (OR: 1.895; 95% CI: 0.580–5.874; *p* = 0.282).

On univariate analysis, vaccine hesitancy was found to be significantly less in respondents between the ages of 30 and 59 years and ≥60 years, people who have been living in the US for longer than 20 years, people who had a professional degree, and people who personally knew someone who had died of COVID-19. However, these associations were not statistically significant on multivariable analysis. A summary of the univariate and multivariable analysis is provided in Table 3.

In this study, the answer “will probably get the vaccine” was categorized into the vaccine hesitant group. To investigate the influence of this categorization, the answer “will probably get the vaccine” was regrouped into “no vaccine hesitancy” and the corresponding data set was reanalyzed. The multivariable logistic regression analysis showed that the overall conclusions are consistent with Table 3 except for “What is your yearly household income?”, which was not significant, but the trend remained the same (Supplemental Table S1).

## 4. Discussion

Our study is unique because it provides important information about the attitudes and beliefs surrounding COVID-19 vaccination of a subgroup of AA, a rapidly growing ethnic minority in the US. Our focus on AA that are employed (or training to be) in the medical field represents a group of individuals that will be crucial in working with the general population, as well as the AA population as part of the COVID-19 vaccination advocacy efforts in the US. Vaccine hesitancy is an urgent public health problem that is unfortunately more prevalent in minority groups [22,23,24]. An enormous hurdle to overcome among AA is the misinformation surrounding vaccines, especially when combined with several conspiracy theories that circulate on social media [11]. Thus, trust-building and regular assessments of vaccine hesitancy will be crucial to achieving COVID-19 vaccine compliance.

From our survey, we found that participants who did not receive an influenza vaccine in the last 5 years were significantly more likely to be vaccine hesitant after adjusting for other potential factors. Our findings agree with other studies in which individuals who previously received an influenza vaccine were more likely to also receive the COVID-19 vaccine [13]. Among healthcare workers, prior influenza vaccine behaviors were an independent predictor for COVID-19 vaccine intentions [25]. This suggests that prior vaccine behaviors, even before the COVID-19 pandemic, influence future vaccine behaviors as well. This may imply that those who deferred prior vaccinations may in fact hold strong vaccine beliefs (“anti-vaccination”), and thus may be unlikely to change despite education on the consequences of not vaccinating [26]. Of note, amongst our cohort of healthcare providers, just 3% of respondents reported prior vaccine behavior of delaying their children’s immunizations, which is low compared to the general population [27]. As the world moves toward childhood immunizations for COVID-19, the relationship between vaccine hesitancy for oneself vs. for one’s child is a topic that should be explored in future studies, particularly among minority groups who may be at higher risk [28].

The type of healthcare occupation was a clear factor in vaccine hesitancy and acceptance in our study. Interestingly, being an allied health professional, which included administrative staff and medical assistants, was strongly and significantly associated with vaccine hesitancy. The observation that physicians are more vaccine acceptant compared to nurses and allied health professionals has been noted in a number of other studies [8,14,23,26,29], including one in the Middle East [13]. The reasons for this attitude in this group of professionals are currently unknown, leaving room for further research. Other studies have reported that a lower educational level was significantly associated with less willingness to take the vaccine [14,22], a finding reported with other vaccinations including influenza [30,31].

Furthermore, a household income of over $150,000/year was less likely to be associated with vaccine hesitancy. As almost half of our study respondents were physicians with professional degrees and earnings over $150,000/year, we hypothesize that in addition to additional education, financial stability may have a role to play in accessing credible and scientific information, which may impact attitudes regarding receipt of the COVID-19 vaccine. A recently published systematic review similarly noted that higher healthcare worker income and education were independently associated with COVID-19 vaccine acceptance [15].

Another interesting finding from our study was the fact that men are less likely to be vaccine hesitant when compared with women. Even though this observation was not sustained in multivariable models, this finding is very important for public health purposes, as it has been hypothesized that this attitude may be related to misinformation about the effect of the vaccine on a female’s fertility [22,32]. Furthermore, prior studies have shown higher vaccine confidence levels among male healthcare workers compared to their female counterparts [15,33], a finding that was also noted in a large survey of Arabs in the general population [14].

Trust in the government has been shown to be strongly associated with vaccine acceptance [14,30]. Thus, of particular interest to the authors was whether immigration to the US, as well as timing of immigration, was associated with vaccine hesitancy. Our study found that on univariate analysis, being born in North America or Europe, rather than the Middle East or North Africa, was significantly associated with vaccine hesitancy. Comparatively, respondents who had immigrated to the US over 20 years ago were significantly less likely to be vaccine hesitant. While not significant on multivariable analysis, prior studies have noted that Arabs living outside of their home country [9] or in North America [10] were more likely to be vaccine acceptant than those living in Arab countries. As noted by the WHO SAGE Working Group, vaccine hesitancy rates and reasons vary by global region, and do not remain static within a country over time [34]. This highlights the importance of frequent assessments within countries to determine if a common concern exists that can be addressed through a national or targeted campaign.

Overall, our study reported a vaccine acceptance rate of 75% among Arab American healthcare workers, which is a combination of respondents who said they already received the COVID-19 vaccine (41%) and respondents who said they were definitely going to receive the COVID-19 vaccine (34%). In comparison, studies of healthcare workers from different countries reported varying COVID-19 acceptance rates ranging from 50.5% in Saudi Arabia [35], 76.6% in China [36], 76.9% in France [37], 79.6% in Libya [38], and 80.9% in Canada [33]. The higher vaccine acceptance rate in healthcare workers compared to the general population is likely explained by the higher levels of education and health literacy among the healthcare study participants. Additional studies that specifically address these observations in other demographic groups and the general population are needed to effectively characterize this finding.

Our study has some unique strengths that are worth emphasizing. To our knowledge, this is the first study to explore COVID-19 vaccine attitudes among AA healthcare professionals living in the US. While this study only focused on AA healthcare professionals, it provides some valuable information about attitudes surrounding the COVID-19 vaccine that may be applicable to AA living in the US in general. With limited health data on this growing demographic, we were able to receive more than 500 responses to the surveys sent. The information gathered on the attitudes and perceptions of AA healthcare workers towards the COVID-19 vaccine may be used to plan public health campaigns and programs that specifically targets this ethnic group, with the aim of increasing vaccine acceptability within this demographic. Furthermore, we designed the survey questions to be easily understood by respondents and thus received near-complete responses to the questions asked, with little to no gaps in information.

In spite of the obvious strengths enumerated above, we acknowledge that our study is not devoid of limitations. Despite sending out 4000 surveys, we received just over 500 responses from respondents, which may indicate some level of disinterest or fatigue around the topic of COVID-19 vaccination among this demographic. The use of e-mail for participant recruitment may have further limited the study, yielding a response bias, given it selected out individuals who do not check their e-mail regularly, those with undeliverable mail, and those who may have filtered out e-mail from certain senders. In addition, this study only focused on AA healthcare professionals with a large representation of physicians. Thus, we recognize that while some of the findings may be applicable to the AA population in general, it is also possible that our findings may not be reflective of the entire AA ethnic group or the American population in general, given that our study only examined healthcare professionals. In addition, we acknowledge that there are other confounding variables that our study did not include, such as health conditions among the participants that would make them more hesitant to receive the vaccine. Religion and cultural factors, which have been factors implicated in vaccine hesitancy in the past [32], were also not evaluated in our study. Finally, our study only captured vaccine attitudes at one point in time, whereas it is understood that vaccine hesitancy is fluid and complicated in nature, does not always translate into behaviors [39], and can readily change with time or exposure to new information.

## 5. Conclusions

When addressing the challenge of vaccine hesitancy among minority groups, identifying their source of vaccine information is critical. As people turn to their healthcare providers for trusted information, addressing vaccine hesitancy among clinicians, and particularly among allied health professionals, is of the utmost importance. Our study shows that prior vaccine attitudes, income, and healthcare role have an impact on willingness to receive the COVID-19 vaccine. Therefore, vaccine advocacy campaigns should be carefully crafted to address these factors. Additionally, vaccine hesitancy among health care providers could have substantial impact on vaccine attitudes of the general population. Campaigns should be geared at vaccine education, particularly among those with lower median income and education levels as well as addressing negative emotions including fear, anxiety, and mistrust. These data should inform policy by government ministries to implement vaccine promotion campaigns in different healthcare provider groups.

## Figures and Tables

**Table 1:** Survey Responses.

Survey Question	N = 513 N (%)
**What is your age?**	
18–29 years old	216 (42)
30–39 years old	66 (13)
40–49 years old	52 (10)
50–59 years old	97 (19)
60–69 years old	46 (9)
70+ years old	36 (7)
**Which gender do you identify with?**	
Female	238 (46)
Male	271 (53)
Missing	4 (1)
**When did you move to the United States?**	
Less than 10 years	76 (15)
20 years ago, or longer	218 (42)
Born in the United States	218 (42)
Missing	1 (0)
**What is your highest level of education? If currently enrolled, please note your highest degree received.**	
High school or less than high school	26 (5)
Some college or university	38 (7)
Bachelor’s degree or higher	169 (33)
Professional degree	279 (54)
Missing	1 (0)
**What is your employment status?**	
Employed part or full time	317 (62)
Unemployed	17 (3)
Student	148 (29)
Retired	30 (6)
Missing	1 (0)
**What kind of health care professional are you?**	
Physician	245 (48)
Nursing	5 (1)
Allied profession	93 (18)
Student	141 (27)
Missing	29 (6)
**What is your yearly household income?**	
Under $29,999	78 (15)
$30,000–$74,999	78 (15)
$75,000–$149,999	86 (17)
Over $150,000	260 (51)
Missing	11 (2)

**Table 2:** Participant’s attitudes, knowledge and behavior regarding vaccines.

Survey Question	N = 513 N (%)
**If you have children, have you ever delayed having your child receive an immunization for reasons other than illness or allergy?**	
Yes	17 (3)
No	268 (52)
I do not have children	227 (44)
Missing	1 (0)
**Have you received an influenza vaccine in the last 5 years?**	
Yes	443 (86)
No	57 (11)
Do not know/not sure	13 (3)
**I know someone personally who has contracted COVID-19.**	
Yes	486 (95)
No	27 (5)
**I know someone personally who has died of COVID-19.**	
No	219 (43)
Yes	293 (57)
Missing	1 (0)
**I plan to receive a COVID-19 vaccine.**	
I have already received the vaccine.	212 (41)
Definitely going to receive the vaccine.	176 (34)
Will probably get the vaccine.	65 (13)
Do not know/not sure.	30 (6)
Will probably NOT get the vaccine.	19 (4)
Will definitely NOT get the vaccine.	11 (2)
**I think a COVID-19 vaccine should be mandated.**	
Yes	231 (45)
No	162 (32)
Do not know/not sure	118 (23)
Missing	2 (0)

COVID-19, coronavirus disease 2019.

**Table 3:** Univariate and multivariable logistic regression analyses of risk factors associated with vaccine hesitancy (Yes vs. No, No as reference).

Survey Question		Univariate ^a^			Multivariable ^b^	
	E/N	OR (95% CI)	*p*	E/N	OR (95% CI)	*p*
**What is your age?**			**<*0.001* ^c^**			0.358 ^c^
18–29 years old	72/216	Reference		60/185	Reference	
30–59 years old	46/215	0.547 (0.354, 0.838)	***0.005***	38/193	0.625 (0.251, 1.524)	0.302
60+ years old	7/82	0.198 (0.082, 0.417)	**<*0.001***	5/75	0.371 (0.090, 1.400)	0.145
**Which gender do you identify with?**						
Female	76/238	Reference		62/206	Reference	
Male	48/271	0.461 (0.304, 0.694)	**<*0.001***	41/247	0.963 (0.557, 1.669)	0.893
**What is your marital status?**			***0.003* ^c^**			0.227 ^c^
Single	69/230	Reference		55/196	Reference	
Married	46/261	0.501 (0.327, 0.764)	***0.001***	42/239	1.939 (0.861, 4.485)	0.111
Divorced/separated/widowed	7/19	1.394 (0.516, 3.536)	0.498	6/18	2.794 (0.628, 11.841)	0.174
**What country were you born in?**						
North Africa/Middle East	53/275	Reference		42/251	Reference	
North America/Europe	71/229	1.876 (1.249, 2.832)	***0.002***	61/202	0.999 (0.267, 3.390)	0.998
**When did you move to the United States?**			**<*0.001* ^c^**			0.910 ^c^
Less than 10 years	22/76	Reference		18/65	Reference	
20 years ago or longer	32/218	0.422 (0.228, 0.788)	***0.007***	25/196	0.855 (0.342, 2.168)	0.740
Born in the United States	70/218	1.150 (0.658, 2.055)	0.628	60/192	1.120 (0.325, 4.434)	0.863
**What is your highest level of education? If currently enrolled, please note your highest degree received.**						
No professional degree	91/233	Reference		73/191	Reference	
Professional degree	34/279	0.219 (0.139, 0.338)	**<*0.001***	30/262	0.543 (0.270, 1.073)	0.079
**What kind of health care professional are you?**			**<*0.001* ^c^**			***0.049* ^c^**
Physician/Nursing	28/250	Reference		26/238	Reference	
Allied profession	47/93	7.975 (4.585, 14.140)	**<*0.001***	44/88	2.434 (1.117, 5.319)	***0.025***
Student	38/141	2.904 (1.703, 5.007)	**<*0.001***	33/127	1.320 (0.527, 3.366)	0.554
**What is your yearly household income?**						
Under $150,000	90/242	Reference		77/213	Reference	
Over $150,000	30/260	0.223 (0.139, 0.349)	**<*0.001***	26/240	0.455 (0.243, 0.838)	***0.011***
**Have you received an influenza vaccine in the last 5 years?**						
Yes	77/443	Reference		66/403	Reference	
No	42/57	12.967 (7.037, 25.044)	**<*0.001***	37/50	8.896 (4.315, 19.252)	**<*0.001***
**I know someone personally who has contracted COVID-19.**						
Yes	112/486	Reference		94/433	Reference	
No	13/27	3.099 (1.419, 6.730)	***0.005***	9/20	1.895 (0.580, 5.874)	0.282
**I know someone personally who has died of COVID-19.**						
No	64/219	Reference		53/196	Reference	
Yes	61/293	0.638 (0.425, 0.955)	***0.029***	50/257	0.977 (0.556, 1.717)	0.934

COVID-19, coronavirus disease 2019; E/N, numbers of events and participants; OR, odds ratio. Bold and italic is used to emphasize statistical significance (*p* ≤ 0.005). ^a^ Univariable logistic regression analysis. ^b^ Multivariable logistic regression analysis. ^c^ Global *p*-value obtained by likelihood ratio tests.

## Supplementary Materials

The following are available online at https://www.mdpi.com/article/10.3390/vaccines9090942/s1, Table S1: Univariable and multivariable logistic regression analyses of risk factors associated with vaccine hesitancy (Yes vs. No, No as reference), where “Will probably get the vaccine” was considered ‘No’.
