# Supplementary material for: Evaluation of COVID-19 Vaccine Attitudes among Arab American Healthcare Professionals Living in the United States
Source: Vaccines (Basel). 2021 Aug 24;9(9):942. doi: 10.3390/vaccines9090942 (PMC8471462; doi:10.3390/vaccines9090942)
Supplement: Supplementary file 1 [file vaccines-09-00942-s001.zip › vaccines-1277614-supplementary-2.pdf]

Supplemental Table S1. Univariable and multivariable logistic regression analyses of risk factors associated with vaccine hesitancy (Yes vs. No, No as reference), where "Will probably get the vaccine" was considered 'No'

Survey Question	Univariable[a]			Multivariable[b]		
	E/N	OR (95% CI)	p	E/N	OR (95% CI)	p
**What is your age?**			***0.030[c]***			0.576[c]
18-29 years old	32/216	Reference		24/185	Reference	
30-59 years old	26/215	0.791 (0.451, 1.377)	***0.408***	20/193	0.767 (0.254,2.261)	0.633
60+ years old	2/82	0.144 (0.023,0.490)	***0.009***	1/75	0.277 (0.013,2.316)	0.294
**Which gender do you identify with?**						
Female	38/238	Reference		29/206	Reference	
Male	21/271	0.442 (0.248,0.77)	***0.005***	16/247	0.883 (0.401,1.934)	0.756
**What is your marital status?**			0.135[c]			0.214[c]
Single	30/230	Reference		21/196	Reference	
Married	23/261	0.644 (0.359,1.141)	0.134	21/239	2.296 (0.826,6.552)	0.114
Divorced/separated/widowed	4/19	1.778 (0.482,5.290)	0.334	3/18	3.914 (0.545,24.405)	0.152
**What country were you born in?**						
North Africa/Middle East	26/275	Reference		19/251	Reference	
North America/Europe	33/229	1.612 (0.935,2.806)	0.087	26/202	1.249 (0.187,6.890)	0.808
**When did you move to the United States?**			***0.021[c]***			0.164[c]
Less than 10 years	12/76	Reference		10/65	Reference	
20 years ago or longer	15/218	0.394 (0.176,0.900)	***0.024***	10/196	0.315 (0.091,1.056)	0.062
Born in the United States	32/218	0.918 (0.456,1.952)	0.815	25/192	0.465 (0.085,3.303)	0.407
**What is your highest level of education? If currently enrolled, please note your highest degree received.**						
No professional degree	45/233	Reference		32/191	Reference	
Professional degree	15/279	0.237 (0.125,0.429)	***<0.001***	13/262	0.711 (0.258,1.872)	0.498
**What kind of health care professional are you?**			***<0.001[c]***			***0.017[c]***
Physician/Nursing	12/250	Reference		11/238	Reference	
Allied profession	28/93	8.544 (4.210,18.323)	***<0.001***	25/88	3.522 (1.191,10.746)	***0.024***
Student	11/141	1.678 (0.710,3.933)	0.230	9/127	1.164 (0.296,4.648)	0.828
**What is your yearly household income?**						

Under $150,000	43/242	Reference		33/213	Reference	
Over $150,000	15/260	0.283 (0.148,0.514)	***<0.001***	12/240	0.791 (0.317,1.927)	0.608
**Have you received an influenza vaccine in the last 5 years?**						
Yes	28/443	Reference		22/403	Reference	
No	28/57	14.310 (7.543,27.528)	***<0.001***	23/50	8.674 (3.941,19.302)	***<0.001***
**I know someone personally who has contracted COVID-19.**						
Yes	54/486	Reference		42/433	Reference	
No	6/27	2.286 (0.810,5.602)	0.088	3/20	0.480 (0.075,2.414)	0.400
**I know someone personally who has died of COVID-19.**						
No	31/219	Reference		23/196	Reference	
Yes	29/293	0.666 (0.387,1.114)	0.140	22/257	0.810 (0.367,1.772)	0.597

COVID-19, coronavirus disease 2019; E/N, numbers of events and participants; OR, odds ratio. Bold and italic is used to emphasize statistical significance ($p \leq 0.005$). [a]Univariable logistic regression analysis. [b]Multivariable logistic regression analysis. [c]Global p-value obtained by likelihood ratio tests.
